# A Novel Loop: Mutual Regulation Between Epigenetic Modification and the Circadian Clock

**DOI:** 10.3389/fpls.2019.00022

**Published:** 2019-01-29

**Authors:** Shenxiu Du, Liang Chen, Liangfa Ge, Wei Huang

**Affiliations:** ^1^State Key Laboratory for Conservation and Utilization of Subtropical Agro-Bioresources, College of Life Sciences, South China Agricultural University, Guangzhou, China; ^2^Department of Grassland Science, College of Forestry and Landscape Architecture, South China Agricultural University, Guangzhou, China

**Keywords:** circadian clock, epigenetic, acetylation, methylation, phosphorylation, Arabidopsis

## Abstract

In response to periodic environmental fluctuations generated by the rotation of the earth, nearly all organisms have evolved an intrinsic timekeeper, the circadian clock, which can maintain approximate 24-h rhythmic oscillations in biological processes, ultimately conferring fitness benefits. In the model plant Arabidopsis, the core mechanics of the circadian clock can be described as a complex regulatory network of three feedback loops composed of core oscillator genes. Transcriptional regulation of each oscillator gene is necessary to maintain the structure of the circadian clock. As a gene transcription regulatory mechanism, the epigenetic modification of chromatin affects the spatiotemporal expression of multiple genes. Accumulating evidence indicates that epigenetic modification is associated with circadian clock function in animals and plants. In addition, the rhythms of epigenetic modification have a significant influence on the timing of molecular processes, including gene transcription. In this review, we summarize recent progress in research on the roles of histone acetylation, methylation, and phosphorylation in the regulation of clock gene expression in Arabidopsis.

## Epigenetic Regulation of the Circadian Clock

The circadian clock is a ubiquitous molecular oscillator that provides basic timing information and regulates biochemical, physiological, and behavioral processes. In plants, the circadian clock regulatory mechanism regulates responses to the environment at the transcriptional level as well as at physiological and biochemical levels. This rhythmic oscillation of nearly 24 h decreases the unnecessary consumption of energy and organics, while increasing competitive productivity and viability.

Multiple interlocked transcriptional feedback loops formed by transcription factors are central to circadian clock function. In the model plant Arabidopsis, the circadian clock system can be described as a complex regulatory network of three loops. The core loop is composed of three important genes, namely, *CIRCADIAN CLOCK ASSOCIATED 1* (*CCA1*), *LATE ELONGATED HYPOCOTYL* (*LHY*), and *TIMING OF CAB EXPRESSION 1* (*TOC1*). In this loop, CCA1 and LHY inhibit the expression of *TOC1*, whereas TOC1 directly represses *CCA1* and *LHY*, thereby establishing a complete regulatory process. As a DNA-binding transcription factor, TOC1 binds directly to the promoters of *CCA1* and *LHY* to repress their expression. CCA1 and LHY, two MYB transcription factors that are active in the morning of the subjective day, repress the expression of *PSEUDO RESPONSE REGULATOR5*, *7*, and *9* (*PRR5*, *7*, and *9*), whereas PRR5, 7, and 9 in turn suppress the expression of *CCA1* and *LHY*. The evening complex (EC) includes three other key clock components, namely, LUX ARRHYTHMO, EARLY FLOWERING3 (ELF3), and EARLY FLOWERING4 (ELF4). The EC complex can directly repress *PRR9*. In the evening loop, TOC1 suppresses the expression of *GIGANTEA* (*GI*), and GI promotes *TOC1* expression, whereas the transcription of *GI* is inhibited by CCA1 and LHY. These three negative feedback loops, together with the input–output pathway of the circadian clock, constitute a complex regulatory network that controls various physiological and crucial metabolic processes in plants (Huang et al., 2012; Nagel and Kay, 2012; Aguilar-Arnal and Sassone-Corsi, 2015; Oakenfull and Davis, 2017).

The nucleosome is a repeating unit of chromatin fiber that consists of 147 base pairs (bp) of genomic DNA wrapped around an octamer of histones. A standard octamer of histones comprises two copies of each of the four canonical histone proteins: H2A, H2B, H3, and H4. Each histone possesses a highly basic N-terminal tail, which protrudes from the surface of the histone octamer and serves as a substrate for several enzymes that lead to different post-translational modifications, including acetylation, phosphorylation, and methylation. Since histone post-translational modification constitutes an extra (*epi*) layer of gene regulation beyond that of the DNA sequence, this mechanism is termed epigenetic. Epigenetic regulation is necessary for survival and reproduction in unpredictable environments (Wang et al., 2016).

Recent studies have indicated that circadian oscillations in plants need to be monitored to facilitate the modification of oscillator regulatory mechanisms according to circumstances. Interestingly, some of the transgenerational plasticity of the plant circadian clock does not involve the alteration of clock gene DNA sequences, but instead manifests as reversible changes in the chromatin structure that determines the expression of the core oscillator genes. Chromatin reshaping depends on epigenetic factors, such as histone post-translational modifications/replacements, which create a flexible loop of gene regulation (Henriques and Mas, 2013; Baerenfaller et al., 2016; Kim et al., 2017; Hung et al., 2018; Lee and Seo, 2018; Stevenson, 2018).

Here, we provide examples of clock gene regulation mediated via epigenetic alteration, and also discuss rhythmic epigenetic changes in plants as well as the contribution of circadian clock epigenetic modification to the processes of adaptation and acclimation in plants.

## Epigenetic Modifications Regulate the Core Oscillators

In Arabidopsis, the circadian clock is composed of three interlocking transcription–translation feedback loops. The central loop, referred to as the core oscillator, was first proposed a decade ago. This loop comprises the three transcription factors *TOC1*, *CCA1*, and *LHY*. The morning-expressed CCA1 and LHY inhibit the transcription of the evening gene *TOC1*. Conversely, at dusk, TOC1 represses the transcription of *CCA1* and *LHY* (Huang et al., 2012; Oakenfull and Davis, 2017).

Previous studies have shown that histone acetylation, methylation, and phosphorylation are associated with transcriptional regulation of the core oscillator genes in the circadian clock.

## Epigenetic Modifications in the Core Loop

Expression of the circadian clock oscillator gene *TOC1* is modulated by dynamic changes in histone deacetylation in the *TOC1* promoter at dawn. The morning transcription factor CCA1 represses the expression of *TOC1* by binding to the *TOC1* promoter, which is accompanied by conditions favoring histone deacetylation in the *TOC1* promoter (Ni et al., 2009; Huang et al., 2012; Nagel and Kay, 2012).

Histone deacetylase (HDAC) is responsible for this histone deacetylation, which contributes to declining *TOC1* expression near dusk. In a *cca1*/*lhy* double mutant, histone H3 acetylation (H3ac) in the *TOC1* promoter was observed to be higher than that in the wild type, indicating that CCA1 has a strong inhibitory effect on *TOC1* expression and that it antagonizes H3ac to decrease the abundance of *TOC1* mRNA (Ni et al., 2009; Malapeira et al., 2012; Ng et al., 2017).

Characterization of H3ac dynamics in the *TOC1* promoter revealed an interesting regulatory mechanism. Studies examining *CCA1*-overexpressing lines indicated that a decrease in H3ac is associated with the repression of *TOC1*, whereas analysis of a *cca1*/*lhy* double mutant revealed an increase in H3ac in the *TOC1* promoter. These observations indicate that CCA1 represses *TOC1* expression by binding to the TOC1 promoter. In addition, the rhythms of histone H3 deacetylation have been found to be negatively correlated with *TOC1* transcript levels. HDACs can remove acetyl groups on lysine residues, thereby generating hypoacetylated histones, which promote chromatin fiber compaction and gene repression. In plants treated with the HDAC inhibitor trichostatin A, *TOC1* is more highly expressed after dusk (Perales and Mas, 2007; Malapeira et al., 2012), thereby indicating that the declining phase of *TOC1* is induced by HDAC activity. These results also suggest that CCA1, as a repressor of *TOC1*, might rely, at least in part, on the recruitment of HDACs to the *TOC1* promoter (Henriques and Mas, 2013; Barneche et al., 2014).

A further component contributing to chromatin modification in the *TOC1* promoter is REVEILLE 8/LHY-CCA1-LIKE 5 (RVE8/LCL5), which affects the repression of *TOC1*. Similar to *CCA1* and *LHY* transcription, *RVE8* transcription peaks in the morning. Altered expression of RVE8/LCL5 in plants modifies the circadian period. Similar to CCA1, RVE8/LCL5 regulates the expression of *TOC1* by binding to the *TOC1* promoter; however, once bound, it promotes hyperacetylation of H3 in the *TOC1* promoter and subsequently activates the expression of this gene. In contrast, CCA1 inhibits the expression of *TOC1* by promoting histone deacetylation. Thus, although their sequences and expression peaks are similar, *RVE8/LCL5* and *CCA1* have contrasting effects on the regulation of *TOC1* transcription (Farinas and Mas, 2011; Barneche et al., 2014; Horak and Farre, 2015).

Recent studies have shown that the rhythm of histone H3K4 trimethylation (H3K4me3) is related to the oscillatory expression of the core clock genes. Analysis of the correlation of clock gene expression in seedlings treated with an H3K4me3 inhibitor has revealed that, compared with the control seedlings, the circadian rhythms of *CCA1* and *TOC1* display a longer period of expression. Therefore, it is conceivable that H3K4me3 is required to ensure correct expression peaks of the clock genes. The oscillatory waveform of H3K4me3 accumulation in core oscillator gene promoters has been shown to have a phase delay compared with that of H3ac, indicating that H3K4me3 might have a different regulatory mechanism whereby it regulates the expression of clock genes (Perales and Mas, 2007; Ni et al., 2009; Barneche et al., 2014).

The successive accumulation of H3ac (H3K56ac and H3K9ac), H3K4me3, and H3K4me2 is known to exhibit circadian rhythmicity. The inhibition of acetylation and H3K4me3 suppresses the expression of the clock genes. Blocking H3K4me3 enhances the binding activity of circadian clock inhibitors, indicating that H3K4me3 could be a marker of the transformation from activation to inhibition. Specifically, the histone methyltransferase SET DOMAIN GROUP 2/ARABIDOPSIS TRITHORAX-RELATED 3 (SDG2/ATXR3) may directly or indirectly contribute to oscillatory gene expression and H3K4me3 accumulation, and altered expression of *SDG2*/*ATXR3* has been observed to modify the binding activity of certain clock repressors (Malapeira et al., 2012; Henriques and Mas, 2013; Barneche et al., 2014).

LYSINE-SPECIFIC DEMETHYLASE 1-LIKE 1 (LDL1) and LDL2 interact with CCA1 and LHY to repress the expression of *TOC1*. Chromatin immunoprecipitation sequencing (ChIP-Seq) analysis has shown that many circadian genes regulated by CCA1 are targeted by LDL proteins. LDL1 and LDL2 interact with the histone deacetylase HDA6, and the LDL1-HDA6 complex binds directly to the *TOC1* promoter and represses *TOC1* expression by increasing histone deacetylation and H3K4 demethylation. These findings have contributed to elucidating a pathway through which histone modifications regulate clock genes and the inner network of core oscillator genes (Hung et al., 2018).

## Epigenetic Modifications of the Other Loops

The rhythmic expression of the core oscillator gene *TOC1* is preceded by the oscillation of H3ac accumulation in its promoter. Moreover, H3ac accumulation parallels the expression of almost all circadian clock components, including *CCA1*, *LHY*, *PRR9*, *PRR7*, *LUX*, and *TOC1*. In this regard, chromatin immunoprecipitation quantitative PCR (ChIP-qPCR) analysis has revealed that H3K9ac, H3K14ac, and H3K56ac are involved in the transcriptional activation of clock genes (Perales and Mas, 2007; Malapeira et al., 2012).

Recent studies have also revealed that *HISTONE ACETYLTRANSFERASE OF THE TAFII250 FAMILY 2 (HAF2)* is involved in circadian clock regulation. The HAF2 protein facilitates H3ac accumulation in the *LUX* and *PRR5* promoters to activate gene expression at midday, with the expression of *HAF2* being regulated by CCA1. Future studies are expected to further elucidate the HAF2-mediated temporal coordination of late-day and evening-expressed genes (Lee and Seo, 2018).

The expression of *JUMONJI C DOMAIN-CONTAINING* 5 (*JMJD5/JMJ30*), which peaks in the evening, is regulated by the circadian rhythm. In addition, the regulation of JMJD5/JMJ30 is jointly controlled by CCA1 and LHY. LHY can suppress the expression of *JMJD5/JMJ30* by directly binding to the *JMJD5/JMJ30* promoter. Furthermore, the expression of *CCA1* and *LHY* under high-intensity red light has been found to be lower in a *jmjd5/jmj30* mutant than in the wild type, indicating that JMJD5/JMJ30, CCA1, and LHY form a negative feedback loop in response to red light (Jones et al., 2010).

Although histone H3 phosphorylation is known to play a role in the regulation of gene transcription, there have been few reports regarding the function of histone H2A phosphorylation in the promoters of circadian clock genes. Recent work has, nevertheless, demonstrated that MUT9P LIKE KINASE4 (MLK4) induces *GI* expression (Su et al., 2017). In this process, MLK4 initially interacts with CCA1 at the *GI* promoter. CCA1 in turn recruits YAF9a, resulting in accumulation of the histone variant H2A.Z and the acetylation of H4 at *GI*, thereby inducing *GI* expression (Su et al., 2017).

The monoubiquitination of histone H2B (H2Bub) is widely observed in plant clock genes, and H2Bub has been shown to have substantial effects on the oscillatory expression of circadian clock genes. The loss-of-function mutant *histone mono-ubiquitination1* (*hub1-1)* exhibits reduced H2Bub accumulation. The oscillation of *CCA1* and *ELF4* is dampened in *hub1-1*, and the LHY expression phase is also advanced. Moreover, *hub1-1* mutation may enhance the expression of *TOC1* by reducing the inhibitory activity of CCA1. H2Bub appears to act as a positive regulator of *TOC1*, *PRR7*, and *GI* expression in etiolated seedlings exposed to light. On the basis of evidence obtained to date, it appears that histone H2B may affect a large number of clock components, the mRNA abundances of which are tightly regulated by intense oscillations (Bourbousse et al., 2012; Barneche et al., 2014) (Table 1).

**Table 1:** Chromatin modifications and related factors associated with the expression of clock genes.

Process	Histone mark	Factor	Target gene	Reference
Acetylation	H3ac	nd	*CCA1, LHY, TOC1*	Perales and Mas, 2007
				Hemmes et al., 2012
	H3K9ac	nd	*CCA1, LHY, TOC1, PRR9, PRR7, LUX*	Malapeira et al., 2012
	H3K14ac	nd	*CCA1, LHY, TOC1*	Hemmes et al., 2012
	H3K56ac	nd	*CCA1, LHY, TOC1, PRR9, PRR7, LUX*	Malapeira et al., 2012
	H3ac	HAF2	*PRR5, LUX*	Lee and Seo, 2018
Methylation	H3K4me3	SDG2/ATXR3	*CCA1, LHY, TOC1, PRR9, PRR7, LUX*	Malapeira et al., 2012
	H3K4me2	nd	*CCA1, LHY, TOC1*	Song and Noh, 2012
	H3K36me2	JMJD5/JMJ30	nd	Jones et al., 2010
Monoubiquitination	H2Bub	HUB1	*CCA1, ELF4*	Himanen et al., 2012
Deacetylation	H3ac deacetylation	HDAC (nd)	*CCA1, LHY, TOC1, PRR9, PRR7, LUX*	Malapeira et al., 2012
	H3ac deacetylation	HDA6	*TOC1*	Hung et al., 2018
Demethylation	H3K4me2	LDL1/LDL2	*TOC1*	Hung et al., 2018
Phosphorylation	H2A Serine 95	MLK4	*GI*	Su et al., 2017

DET1 may act as a transcriptional corepressor of CCA1 and LHY to repress *TOC1* transcription. Similar to *cca1/lhy* mutants, a *det1-1* mutant was shown to exhibit a shorter period of *TOC1* oscillations. Given that DET1 interacts with H2Bub, it may repress clock genes via H2Bub (He et al., 2011; Lau et al., 2011; Kang et al., 2015). A further study has revealed that PRR5, 7, and 9 can interact with *TOPLESS/TOPLESS RELATED PROTEINS (TPL/TPR)* and *HDA6* to form a complex at the promoters of *CCA1* and *LHY*, thereby repressing the expression of these two genes (Wang et al., 2010, 2013).

## The Rhythmic Expression of Histone-Modification Enzymes

Histone modifications, including acetylation–deacetylation and methylation–demethylation, play a key role in regulating the expression of clock genes, and in this regard, previous studies have indicated that the rhythmic expression of epigenetic enzymes is correlated with the daily rhythms of epigenetic modification and the expression of downstream genes (Loenen and Raleigh, 2014; Baerenfaller et al., 2016).

## Histone Deacetylases

Histone acetylation/deacetylation modifications are important for gene transcription, and the oscillatory expression of clock genes is known to be associated with such modifications. Although evidence indicates that HDACs can directly regulate the expression of clock genes, the effects of individual HDAC genes have yet to be elucidated. Currently, the function of HDACs is generally studied by treatment with HDAC-specific inhibitors suberoylanilide hydroxamic acid (SAHA), trichostatin A, or butyrate or by the overexpression and repression of HDAC genes in transgenic plants. Plant HDAC family can be divided into three subfamilies, namely, the *HISTONE DEACETYLASE 1 (HDA1)*, *SIRTUIN 2 (SIR2)*, and *HISTONE DEACETYLASE 2 (HD2)* subfamilies. In Arabidopsis, members of the HDA1 subfamily include *HDA2*, *5*, *6*, *7*, *8*, *9*, *10*, *14*, *15*, *17*, *18*, and *19*, whereas the SIR subfamily includes *STR1* and *STR2*, and the *HD2* subfamily includes *HDT1*, *2*, *and 3* (Hollender and Liu, 2008; Wang et al., 2014; Bourque et al., 2016). Among the HDACs in the Diurnal database of the Mockler Laboratory^1^, *HD2B*, *HDA2*, *HDA4*, *HDA5*, *HDA6*, *HDA7*, *HDA8*, *HDA9*, *HDA17*, *HDA18*, *HDA19*, *SRT1*, and *SRT2* are rhythmically expressed (Table 2), with the expression of *HDA4*, *5*, *6*, *7*, *9*, *10*, *17*, and *18*, and *HD2B*, *SRT1*, and *SRT2* peaking at midnight, and that of *HDA2* and *8* peaking at midday (Diurnal database).

**Table 2:** The rhythmic expression of epigenetic modification enzymes.

Name	Locus	Light condition	Phase	Correlation	Reference
HDA6	AT5G63110	LL	22	0.65049	Diurnal
HDA9	AT3G44680	LL	20	0.87604	Diurnal
HDA2	AT5G26040	LL	12	0.89002	Diurnal
HD2B	AT5G22650	LL	20	0.90944	Diurnal
HDA19	AT4G38130	LL	21	0.90467	Diurnal
HDA17	AT3G44490	LL	20	0.82185	Diurnal
SRT1	AT5G55760	LL	20	0.92113	Diurnal
SRT2	AT5G09230	LL	0	0.8815	Diurnal
HDA8	AT1G08460	LL	10	0.94022	Diurnal
SDG2	AT4G15180	LL	12	0.82954	Diurnal
SDG29/ATX5	AT5G53430	LL	5	0.81682	Diurnal
SDG23/SUVH6	AT2G22740	LL	8	0.9162	Diurnal
SDG4	AT4G30860	LL	17	0.74943	Diurnal
JMJD5	AT3G20810	LL	14	–	Jones et al., 2010

## Histone Methyltransferases

Methylation of lysine residues in the H3 histone tail is a key mechanism contributing to the regulation of chromatin state and gene expression, and is mediated by a family of enzymes with a SET domain. One of the main functions of these enzymes is to regulate H3K4 di- and tri-methylation, which has been discovered in the *TOC1* promoter and shown to play a role in the repression of *TOC1* by CCA1 (Sanchez et al., 2010; Malapeira et al., 2012).

The SET DOMAIN GROUP (SDG) protein family in Arabidopsis contains 49 members and can be divided into five classes based on activity and structure. The five members in class III SDG, *ATX1–5*, which are homologous to the Trithorax proteins in other eukaryotes, have been shown to participate in H3K4me. Among these proteins, ATX1 (SDG27) is important for the trimethylation of H3K4. Although *ATX2/SDG30* shows sequence homology to ATX1, it exhibits H3K4me2 rather than H3K4me3 methylation activity, whereas ATX3/SDG14, ATX4/SDG16, and ATX5/SDG29 have been observed to affect 1000s of H3K4me2 and H3K4me3 sites across the entire Arabidopsis genome.

The SDG family of Arabidopsis also contains seven ATX-related (ATXR) proteins, among which ATXR7/SDG25 and ATXR3/SDG2 have functions similar to that of ATX. The function of ATXR3/SDG2 is comparable to that of ATX3/SDG14, ATX4/SDG16, and ATX5/SDG29, and it may regulate clock gene expression by modulating H3K4me3 in promoters. However, unlike the expression of *ATX5/SDG29*, which peaks in the morning, peak expression of *ATXR3/SDG2* occurs at midday. The Diurnal database indicates that both *ATXR3/SDG2* and *ATX5/SDG29* have rhythmic expression (Table 2) (Malapeira et al., 2012; Chen et al., 2017).

The other three important SDG proteins, SU(VAR)3-9 HOMOLOG 4 (SUVH4), SUVH5, and SUVH6, are histone H3 lysine 9 (H3K9) methyltransferases. SUVH4 and SUVH6 are responsible for maintaining the H3K9 methylation of inverted repeats during transcription, whereas SUVH5 is necessary for the accumulation of H3K9me2 DNA methylation. Recent studies have shown that HDA6 can interact with these three histone methyltransferases, and indicate that the C-terminal region of HDA6 is important for this interaction. In this regard, two phosphorylated serine residues, S427 and S429, have been identified in the C-terminal region of HDA6, and HDA6 phosphorylation (amino acid substituents that mimic phosphorylated proteins) has been observed to lead to increased enzyme activity. Furthermore, mutation of S427 in HDA6 to alanine was found to abolish the interactions between HDA6 and SUVH5 and SUVH6, thereby indicating that the phosphorylation of HDA6 is important for its activity and function (Yu et al., 2017). The ChIP-seq result also showed that the SUVH members displayed different DNA binding preferences, deciphering the mechanism of sequence-biased non-CG methylation in plant methylomes (Li et al., 2018).

Currently, the involvement of *SUVH4*, *5*, and *6* in the circadian clock is largely unknown; however, a previous study has shown that SUVH4, 5 and 6 affect H3K9me but not H3K4me in the *TOC1* promoter. According to the Diurnal database (Table 2), *SUVH4*, *5*, and *6* and *HDA6* are rhythmically expressed, and given that SUVH4, 5 and 6 interact with HDA6, it is probable that they play a role in circadian clock regulation (Cho et al., 2012).

## Histone Demethylases

Recent studies have demonstrated that histone methylation can be inhibited by at least two different types of enzymes, LSD1 and the JMJ proteins. As discussed above, JMJD5/JMJ30 is a component of the circadian clock in Arabidopsis, and the expression of *JMJD5/JMJ30* peaks 2 h after midday (Jones et al., 2010; Hemmes et al., 2012). A further two JMJ proteins, JMJ20 and JMJ22, have also been shown to be involved in the regulation of clock genes. When the important clock input pathway gene phytochrome B (PHYB) is inactive, JMJ20 and JMJ22 are directly repressed by the zinc-finger protein SOMNUS (Lu et al., 2008; Cho et al., 2012). The Diurnal database indicates that *JMJ22* is rhythmically expressed and that its expression peaks in the evening (Table 2).

## Conclusion and Perspectives

Epigenetic regulation of the circadian clock has recently been investigated via advanced molecular biology and genetic approaches. The periodic expression of core clock genes is regulated epigenetically at the chromatin level and modification of histones primarily leads to alterations in the transcriptional activity of clock genes. Interestingly, the deacetylation of H3ac is related to H3K4me demethylation, which directly connects histone acetylation with methylation. In addition, the phosphorylation of H2A by MLK4 directly regulates the formation of H2A.Z and the acetylation of H4. These observations indicate that epigenetic regulation plays an important role in the regulation of the circadian clock. We also highlight that certain epigenetic modification enzymes have a rhythmic expression, suggesting that clock genes may regulate epigenetic modification enzymes (Su et al., 2017).

**FIGURE 1:**
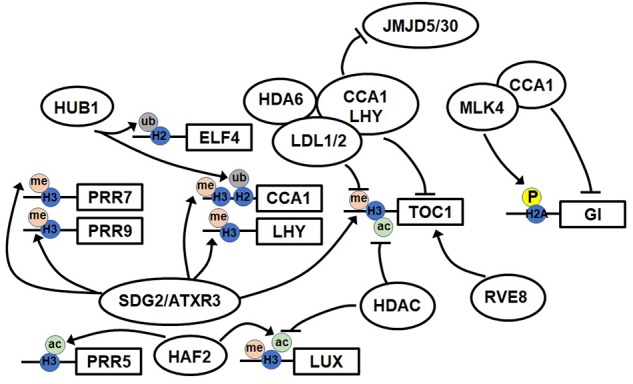
Model of the epigenetic regulation of clock genes. The model shows known epigenetic modifications at the promoters of clock genes and factors. The accumulation of histone H3 acetylation (H3ac), H3 lysine 3 methylation (H3K4me3), and histone H2 ubiquitination (H2ub) induces the expression of clock genes. The phosphorylation of histone H2A inhibits the accumulation of histone variant H2A.Z and also the acetylation of H4.

In addition, histone acetylation modification is a reversible dynamic process that involves both HDACs and HISTONE ACETYLTRANSFERASES (HATs). Although HAF2 is known to regulate PRR5 and LUX (Lee and Seo, 2018), the involvement of other HATs with regards to circadian rhythms remains unclear (Aquea et al., 2017). In the Diurnal database for Arabidopsis, we found that peak expression of *HISTONE ACETYLTRANSFERASE OF THE GNAT 5* (*HAG5*), *HISTONE ACETYLTRANSFERASE OF THE CBP FAMILY 12* (*HAC12*), *HAC1*, *HAC12, HAF1, HAC2*, *HAC4*, and *HAC5* occurs in the morning, whereas peak expression of *HAG2* and *HAG3* occurs in the evening, and only the expression of *HAC1* peaks near midday (Wang et al., 2014; Fina et al., 2017) (Figure 1).

Accumulating evidence gained from studies on the mammalian showed that the epigenetic modification is also important for the mammalian circadian clock. Similar to plants, histone acetylation and methylation also regulate the mammalian circadian clock. Consistent with the expression rhythm of clock genes, the histone acetylation H3K9ac and H3K27ac also displays circadian rhythm (Ripperger and Schibler, 2006; Feng et al., 2011; Vollmers et al., 2012). Histone methylation (H3K4me3) rhythmically oscillates at transcription start sites (TSSs) of clock genes (Le Martelot et al., 2012; Yue et al., 2017). In mammalian, the rhythmic expression of DNA methyltransferase indicated that the DNA methylation is involved in the transcription and regulation of clock genes (Benoit et al., 2013; Masri et al., 2015; Ng et al., 2017; Padmanabhan and Billaud, 2017; Kwapis et al., 2018). To date, however, no similar evidence has emerged in plants. Higher plants have three DNA methylation sites, namely, CG, CHG, and CHH (where H is A, C, or T), among which the methylation of CG and CHG sites is most important for the regulation of gene expression. DNA methyltransferases can alter the DNA methylation level of CG and CHG sites (Underwood et al., 2018), and in the Diurnal database for Arabidopsis, we found that *CHROMOMETHYLASE 3* (*CMT3*), *CMT2, DNA METHYLTRANSFE RASE 1* (*MET1*), *MET2*, *DORMANCY-ASSOCIATED PROTEIN 1* (*DRM1*), and *DRM2* have rhythmic expression patterns, with peak expression of DRM1 and DRM2 occurring near midday, that of *CMT3* and *MET1* occurring in the evening, and only that of *CMT2* occurring near midnight (Diurnal database; Xia, 2008; Wang et al., 2014).

**FIGURE 2:**
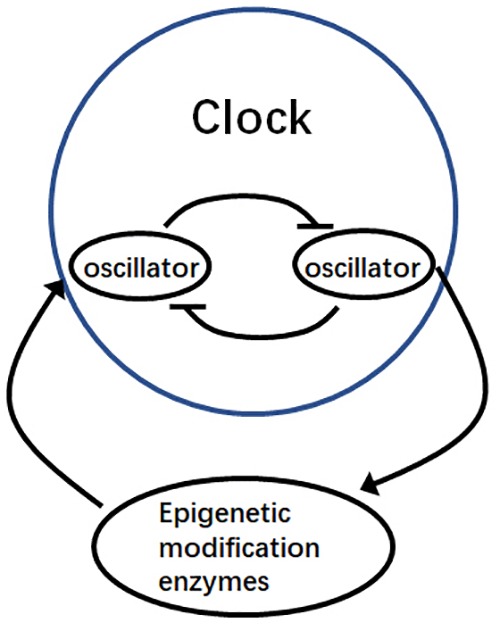
Mutual regulation via epigenetic modification and the circadian clock. Oscillator genes regulate the expression of epigenetic modification enzymes, which in turn influence the regulation of other oscillator genes during the circadian cycle.

Although the mechanisms underlying the associations between epigenetic modifications and the circadian clock have recently been a focus of research, the relationships between certain epigenetic modification enzymes and the circadian clock currently remain undetermined. Nevertheless, data obtained from ChIP-Seq analysis of the core oscillator genes in the Arabidopsis circadian clock have indicated that many epigenetic modification enzymes are rhythmically expressed. These findings provide compelling evidence that epigenetic modification enzymes are directly regulated by core oscillator genes. Thus, we hypothesize that the circadian clock can directly regulate epigenetic modification enzymes and that these enzymes in turn contribute feedback to the circadian clock, involving the mutual regulation of core oscillators (Figure 2). This potential output pathway might be an interesting topic of plant circadian clock study in future. The oscillator genes that directly regulate transcriptional epigenetic modification enzymes are yet to be found. The DNA methylation modification of core oscillator genes still needs to be detected. The mechanisms underlying the epigenetic modification of the core circadian clock genes remain to be further elucidated.

## Data Availability

Publicly available datasets were analyzed in this study. This data can be found here: http://www.mocklerlab.org/supplements.

## Author Contributions

WH conceived the article. SD wrote the first draft. LC and LG critically revised the article. All authors were involved in the revision of the drafted manuscript and have agreed to the final content.

## Conflict of Interest Statement

The authors declare that the research was conducted in the absence of any commercial or financial relationships that could be construed as a potential conflict of interest.

## Footnotes

1 http://diurnal.mocklerlab.org/diurnal\_data\_finders/new
